# Clinical Studies that Initiated the Use of Spinal Opioids for the Treatment of Pain: A New Approach to Historical Review

**DOI:** 10.2174/2772432817666220609093243

**Published:** 2023-10-06

**Authors:** Igor Kissin

**Affiliations:** 1 The Department of Anesthesiology, Perioperative and Pain Medicine, Harvard Medical School, Brigham and Women's Hospital, Boston, Massachusetts, 02115 USA

**Keywords:** Epidural analgesia, citation impact, clinical trials, intrathecal analgesia, opioids, priority rules

## Abstract

Opioids administered into the spinal space by intrathecal or epidural routes can provide potent and prolonged selective analgesia. Compared to the systemic administration of opioids, spinal administration can bring about analgesia with fewer central and systemic adverse effects. For the past 40 years, spinal opioid analgesia has achieved great popularity in various fields of pain treatment. The aim of this work is to identify clinical studies that initiated the use of spinal opioids for the treatment of pain.

To determine the historical role of each of the review’s studies, we used the combination of two factors: the study priority in terms of the time of its publication and the degree of its acknowledgement in the form of citation impact. The date of publication was regarded as the primary factor, but only if the count of citations indicated a sufficient acknowledgement by the other authors. The citation impact was assessed as the initial citation count – for a period of five years after the year of article publication – and the total count.

Analysis of the related data shows that the clinical studies initiating the use of spinal opioids for the treatment of pain belong to two groups of authors – Wang *et al.* and Behar *et al.* Both studies were published in 1979 and described delivery of morphine into the spinal space, although the techniques of administration were different: Wang *et al.* injected morphine intrathecally, Behar *et al.* administered morphine epidurally. The response to these studies was overwhelming - close to a dozen reports on this topic were published in 1979 and more than a hundred – in 1980-1981. The total citation response to the Wang *et al.* article reached 699, and that to Behar *et al.* – 518. Two earlier records (1900-1901) of the use of intrathecal morphine, by Nicolae Racoviceanu-Pitesti and Otojiro Kitagawa, found no following in medical literature for more than three quarters of a century.

## 1 INTRODUCTION

The discovery that opioids administered into the spinal space by epidural or intrathecal routes can selectively provide potent and prolonged analgesia opened new perspectives in the treatment of pain. Compared to systemic administration of opioids, their spinal (neuraxial) administration provides important clinical advantages. The apparent absence of spinal autonomic, motor, or respiratory systems provides the basis for selective analgesic action of spinal opioids [1]. As a result, spinal (neuraxial) opioids can bring about analgesia with fewer central and systemic adverse effects than opioids administered systemically. For the past 40 years, they achieved great popularity in a variety of settings as sole analgesics or in combination with low-dose local anesthetics [2]. A quantitative history of academic publications related to spinal opioids is shown in Table **1**. It shows that the number of articles representing various fields of spinal opioid analgesia is huge and constantly growing. The use of spinal opioids has become a standard for pain management; they are especially popular in the treatment of postoperative pain and chronic pain, including cancer pain.

The aim of the current work is to identify clinical studies that initiated the use of spinal opioids for the treatment of pain. I also tried to find out what laboratory studies inspired clinicians to perform the initial trials of spinal opioids.

## 2 METHODS

The first reviews of clinical studies on spinal opioids show that close to a dozen reports on this topic were published in 1979 and more than a hundred - in 1980-1981 [3, 4]. One of them mentions a 1977 laboratory article that also briefly reports a trial of intrathecal morphine in human subjects [3]. This information determined the primary time interval for the current search – 1977-1981 - for initial studies demonstrating that opioids, injected intrathecally or epidurally, provide clinically meaningful pain relief.

In science, “priority rule” is the credit given to the person(s) who first published a new finding [5, 6]. Determining priority of discovery is a two-step process that includes its disclosure and validation [7]. The validation reflects the scientific community’s response to a disclosure. In practice, this acknowledgement usually comes in the form of citations in articles by other authors, which can accumulate over time. In the current search, two factors were used in combination: the study priority in terms of the time of the study publication and its acknowledgement in the form of citation impact. The time of the study publication was regarded as a primary factor, but only if its citation count was indicative of sufficient acknowledgement by the authors of other articles.

Searches were conducted mostly by hand from published articles on spinal opioids. In addition, PubMed, Web of Science, and Google Scholar databases were also used. The following terms were placed into the search boxes – the type of administration: spinal, neuraxial, intrathecal, epidural, and extradural; and the name of the agents: opioids, morphine, meperidine, methadone, hydromorphone, fentanyl, and diamorphine. To determine the citation impact of the article, the Web of Science database was used. For each article found, the total citation count and the count for a period of five years after the year of article publication were determined.

## 3 RESULTS

The initial clinical studies reporting the long-lasting analgesic effect of spinal opioids were by two groups of authors – Wang *et al.* [8] and Behar *et al.* [9]. They published their results in 1979 within one month of each other: Wang *et al.* in Anesthesiology in February (accepted for publication on May 9, 1978) and Behar *et al.* in The Lancet in March (published as preliminary communication, though the date of acceptance for publication was not indicated). Although both groups delivered morphine into the spinal space, the technique of administration differed. Wang *et al.* injected morphine (0.5 or 1.0 mg) intrathecally at the L2 – L3 interspaces to eight patients who had intractable cancer pain. Behar *et al.* administered morphine (2 mg) epidurally to 10 patients with severe acute postoperative or chronic pain, at the T7 to L4 interspaces, depending on the pain location. The acknowledgment of these two reports was very impressive and quite similar in degree. As indicated in Table **2**, the article by Wang *et al.* had a total citation count of 699, with 170 during the first five years after publication. The total citation count for the article by Behar *et al.* was 518, 181 of those during the first five years after publication.

In 1979-1980, many other reports confirming the clinical value of spinal opioid analgesia were published in rapid succession. Table **2** presents only those that received more than 200 citations. In the May 1979 Lancet, Cousins *et al.* [10], in a response to the Behar *et al.* article, presented the results of intrathecal or epidural administrations of morphine or meperidine in 18 patients with cancer pain or acute postoperative pain. They reported pharmacokinetic and neurological data supporting the conclusion on a selective spinal analgesic action of opioids. This publication received 205 citations, 94 of them within five years.

In March 1980, the Magora *et al.* group (basically the same group from the Hadassah University Hospital that produced Behar *et al.* [9]) published in the British Journal of Anaesthesiology, an article on the effect of epidural morphine (2-3 mg) in 98 patients with various acute or chronic pain conditions [11]. They concluded that morphine-induced pain relief was good or excellent in 56% of those patients and that there were no motor, sensory or sympathetic blockades and no respiratory or hemodynamic complications. The total citation response to this article was 203, most of which (119) within five years of its publication.

The last article in Table **2** belongs to Bromage *et al.*, who assessed epidural analgesia with opioids in 66 postoperative patients [12]. The authors concluded that epidural opioids (morphine, hydromorphone, or methadone) are effective for production of prolonged and segmental postoperative analgesia. The total citation count of this article reached 398, 81 of which were within the first five years.

Table **3** presents references included in these five articles, some of which (the left side) are on previous studies on humans. Though Wang *et al.* article [8] does not have such references, in the table’s section on animal studies (the right side) there is a reference to Wang’s study on the analgesic effect of intrathecally administered morphine published in July 1977 in Regional Anesthesia [13]. In this article, Wang presented the result of his experiments in rats, but in the discussion section, there is a statement that a clinical trial of intrathecal administration of morphine for intractable cancer pain was underway and the preliminary results were very encouraging. The Behar *et al.* article [9] has a reference to another experimental publication by Wang, also mentioning his preliminary observations on the intrathecal administration of morphine in humans (May 1978, Annales De L Anesthesiologie Francaise) [14].

All other articles presented in Table **3** have references to the clinical studies by Wang *et al.* [8] and Behar *et al.* [9]. In addition, Magora *et al.* [11] and Bromage *et al.* [12] refer to the previously published articles of Cousins *et al.* [10] and Samii *et al.* [15]. The Samii *et al.* article, though one of the first responses (May 1979, The Lancet) to the publication by Behar *et al.* [9], is not included in Table **2**, because it had far fewer citations (total count 76) than other articles. Among multiple responses to the Behar *et al.* article (most published in The Lancet) there are those that first reported cases of respiratory depression after spinal administration of opioids: Scott and McClure [16], Glynn *et al.* [17], and Liolios and Andersen [18].

The main aim of Table **3** was to show which of the studies in animals may have prompted the authors of clinical studies to try spinal opioid analgesia in humans. As expected, such laboratory studies were cited by the authors of clinical studies. With the Wang *et al.* article [8], the answer is very obvious because Josef Wang himself was the sole author of two studies in rats on the analgesic effects of intrathecally injected morphine [13] and serotonin [19]. These studies undoubtedly strongly motivated the author to use intrathecal morphine in humans. The reference list of the Wang *et al.* article [8] has three other references to laboratory studies. One is by Pert, *et al*. on autoradiographic localization of the opioid receptor in the rat brain [20]; two others are on analgesia mediated by the direct spinal action of opioids, both by the Yaksh group [21, 22].

Two types of references to the laboratory studies in the article by Behar *et al.* [9] indicate what was important for their decision to start a clinical trial: the identification of the opioid receptors in the spinal cord – from studies by Snyder [23, 24]; and the demonstration that the intrathecal injection of opioids in animals produces profound analgesia from studies by Yaksh *et al.* [21, 25-27]. Since the epidural has some clinical advantages over the intrathecal route, Behar *et al.* decided to use the former. Table **3** shows that the three other articles (3-5) cited mostly the same animal studies. The most frequently cited were the articles by the Yaksh group and the Snyder group.

When comparing the dates of two categories of notable references in Table **3** [20-34] - those to studies in animals with those to studies in humans - one general feature becomes obvious - a very short time period between them. With the Wang *et al.* [8] and Behar *et al.* [9] articles, the references to animal studies indicate publication dates 1976-1977, and the first human studies were published in 1979. In the case of the clinical study by Wang *et al.* [8], preliminary results were published as early as 1977 [13]. Thus, clinical studies on the use of spinal opioids for pain treatment started quite soon after the most important laboratory studies were published.

The search for studies of the spinal administration of opioids revealed two records of intrathecal administration of morphine close to the clinical trials of intrathecal spinal anesthesia with cocaine, introduced by Bier in 1898 [35]. One is by a Romanian surgeon, Racoviceanu-Pitesti, who in 1900 injected intrathecally a combination of cocaine and morphine [36]. Another record reports that in 1901, the Japanese surgeon, Matsuki described two patients with severe back pain who received an intrathecal injection of a combination of morphine and a local anesthetic (eucaine); both had pain relief lasting for several days [37].

## 4 DISCUSSION

Both the time-line of study publications and their acknowledgement in the form of citation impact show that two groups of authors initiated spinal opioid analgesia: Wang *et al.*, who used intrathecal administration of morphine; and Behar *et al.*, who administered morphine epidurally. Several other studies included in Table **2** (and many others as well), made important contributions to the development of this method of pain treatment. The rapid pace of its development is illustrated by the rise in the number of new studies published on one of the fields of spinal opioid analgesia – treatment of postoperative pain. According to the PubMed database, in 1979-1980 there were 33 academic articles on this topic; in 1981-1982 – 104 articles; and in 1989-1990, the number of articles reached 182. There are two reviews on the history of neuraxial administration of local anesthetics and opioids that presented a general picture of events [38, 39]. Relevant clinical information on the use of spinal opioids in the 1980s-90s was reviewed by Rawal [2] and Morgan [40].

The Wang *et al.* study has some advantages over the Behar *et al.* study in that it was slightly earlier. It is not so much the one-month difference in publications (February 1979 with Wang *et al. vs*. March 1979 with Behar *et al.*) as the much earlier Wang’ publications (in 1977 [13] and 1978 [14]) of preliminary observation of the intrathecal administrations of morphine in humans. The latter of these two publications was cited in the article by Behar *et al.* [9]. One of the coauthors of the Behar *et al.* article, Magora, in 2004 indicated [41] that they reported the spinal effect of morphine after Wang *et al.*

At the same time, the Behar *et al.* study also has some advantages. First, they used the epidural, rather than intrathecal route of administration which has a wider appeal among physicians. In addition, they submitted their manuscript to a journal with a very broad readership – The Lancet. As a result, in 1979 alone, the Behar *et al.* publication elicited six published letters to that journal describing new results of spinal opioid administration in response to Behar’s article. It would be difficult to find a better example of successful placement of a new technique on “the escalator of progress”.

The question of which laboratory studies inspired the authors of the first clinical trials of spinal opioids is answered by the clinical studies’ references. The identification of opioid receptors in the spinal cord [23, 24] and the demonstration of intrathecal opioids analgesia in animals [21, 29] were recognized as important motivating factors in the use of spinal opioids in patients. However, the cited laboratory studies address only the motivational aspects of the story of spinal opioid analgesia, not its many other aspects. The consummate presentation of laboratory studies on spinal opioid analgesia can be found in reviews by Kitahata and Collins [3] and Yaksh [1]. Animal studies were the foundation for the clinical advancement of spinal opioid analgesia.

The other interesting feature in the relationship between laboratory and clinical studies on spinal opioid analgesia is how rapidly laboratory research moved into the clinic. The best illustration is the 1977 article by Wang, when after presenting the results on the analgesic effect of intrathecal morphine in rats, he reports the preliminary results on patients [13]. The extraordinary speed of movement from laboratory to the clinic was well explained by Winnie: there was no requirement for any “central” control, for the drugs or for the techniques, only the application was new [42]. In fact, the timeline of the publications of laboratory and clinical studies on spinal opioids reflects one uninterrupted chain of events.

The current work is based on events in the 1970s and 1980s. The contemporary situation with spinal opioid analgesia can be judged by looking at the numbers of recent academic articles presented in Table **1**. It includes only studies on the most common fields of neuraxial opioid analgesia; there are many other areas in which epidural or intrethecal opioids are used for the treatment of pain [43]. The present status of the clinical pharmacology of opioids delivered by epidural or intrathecal injections is usually reviewed according to the specific area where spinal opioid analgesia is used. For example, the current use of neuraxial opioids for the treatment of postoperative pain is presented by Hurley *et al*. [44] in the Miller’s textbook of Anesthesiology.

In the present assessment, a combination of two factors was used: the study’s priority in terms of the time of its publication and the degree of its acknowledgement by the academic community in the form of citation. This approach is an attempt to introduce more objective evaluation of the study’s historical role in the development of the new treatment modality. It should be regarded as an initial attempt in the assessment of the studies that fomented new developments in pain treatments.

The remarkable thing in the history of spinal opioid analgesia is that physicians, who were recorded as the first to inject morphine intrathecally, are not among those who published studies in 1979. Those records belong to Nicolae Racoviceanu-Pitesti [36] and Matsuki [37] in 1900-1901, more than three quarters of a century earlier. These facts raise the question of precedence in the discovery of spinal opioid analgesia. The decisive factors in the consideration of this question are not only the years of the records, but also the entire context of related events. Wang *et al.* and Behar *et al.* were the first to initiate the real clinical use of neuraxial opioids analgesia, for the following reasons. First, their 1979 publications caused an avalanche of clinical studies that opened new avenue for the treatment of pain. The extremely high citation impact of these 1979 studies reflects their original role in the development of the field. Second, these two studies represent a natural and timely bridge between laboratory discoveries (such as the presence of opioid receptors in the spinal cord and the direct analgesic effect of opioids injected intrathecally in animals) and clinical demonstrations of spinal opioid analgesia.

## CONCLUSION

In conclusion, Wang *et al.* and Behar *et al.* initiated the use of spinal opioids for the treatment of pain; their studies were published in 1979 within a month of each other. Both groups delivered morphine into the spinal space, although the techniques of administration were different: Wang *et al.* injected morphine intrathecally, Behar *et al.* - epidurally. The response was overwhelming, with nearly a dozen reports on the topic published in 1979 and more than a hundred – in 1980-1981. The citation response was also impressive: 170 citations to the Wang *et al.* article, in just the five years following publication, and 181 citations to the Behar *et al.* article.

## Figures and Tables

**Table 1:** PubMed^a^ articles on the use of epidural and intrathecal opioids for the treatment of pain.

**Years**	**Postoperative Pain^b^**		**Chronic Pain^c^**		**Cancer Pain^d^**	
	**Number of Articles**	**PI^e^**	**Number of Articles**	**PI^f^**	**Number of Articles**	**PI^g^**
1981-85	270	9.76	30	1.27	63	6.18
1986-90	386	9.21	47	1.29	108	5.78
1991-95	540	7.97	53	1.02	116	4.07
1996-00	535	5.92	100	1.28	110	2.69
2000-05	588	4.81	133	1.05	97	1.58
2006-10	570	3.24	202	1.06	99	1.14
2011-15	547	2.13	247	0.88	155	1.10
2016-20	686	1.86	221	0.58	204	0.92

**Note: **^a^ PubMed, the search engine of the United States Library of Medicine, it comprises more than 32 million citations from the biomedical literature for medicine, life science journals, and online books.

^b^ Articles found with the combination of the following terms “Pain, Postoperative AND (Epidural opioids OR Intrathecal opioids).”

^c^ Articles found with the combination of the following terms “Chronic Pain NOT Cancer Pain AND (Epidural opioids OR Intrathecal opioids).”

^d^ Articles found with the combination of the following terms “Cancer Pain AND (Epidural opioids and Intrathecal opioids).”

^e^ Popularity Index (PI)^43^ is the percentage of articles on the indicated combination of terms among all articles on postoperative pain.

^f^ The percentage of articles on the indicated combination of terms among all articles on chronic pain.

^g^ The percentage of articles on the indicated combination of terms among all articles on cancer pain.

**Table 2:** Initial high-impact clinical studies on the use of intrathecal and epidural opioids for the treatment of pain.

**S. No.** **#**	**First Author**	**Technique, Type of Pain, and Drug(s)**	**Time of Publication, Journal, and Number of Patients**	**Number of Citations**	
				**Total**	**First 5 Years^a^**
1	Wang JK [8]	-Intrathecal -Cancer Pain -Morphine	-1979 (Feb) -*Anesthesiology* -8 Patients	699	170
2	Behar M [9]	-Epidural -Acute or Chronic Pain -Morphine	-1979 (Mar) -*Lancet* -10 patients	518	181
3	Cousins MJ [10]	-Intrathecal or Epidural -Cancer or Postoperative Pain -Morphine or Meperidine	-1979 (May) -*Lancet* -18 patients	205	94
4	Magora F [11]	-Epidural -Acute or Chronic Pain -Morphine	-1980 (Mar) -*Br J Anaesth* -98 patients	203	119
5	Bromage PR [12]	-Epidural -Postoperative Pain -Morphine, Hydromorphone or Methadone	-1980 (Jul) -*Anesth Analg* -66 patients	398	81

**Note: **^a^ The citation count for the period of five years after the year of article publication (included in the total count).

**Table 3:** Notable articles which led to the high-impact clinical studies on spinal analgesia.

**S. No. #**	**High-Impact Study**	**Notable References**	
		**Previous Studies in Humans**	**Guiding Studies in Animals**
1	Wang *et al*, 1979 [8]	-	Pert *et al*, 1976 [20] Yaksh *et al*, 1976 [21] Yaksh *et al*, 1977 [22] Wang, 1977 [13]
2	Behar *et al*, 1979 [9]	Wang, 1978 [14]^a^	Snyder, 1977 [23, 24] Yaksh *et al*, 1976 [21, 25] Yaksh *et al*, 1977 [26] Yaksh *et al*, 1978 [27]
3	Cousins *et al*, 1979 [10]	Wang *et al*, 1979 [8] Behar, 1979 [9]	Yaksh *et al*, 1976 [21] Yaksh *et al*, 1977 [26] Yaksh *et al*, 1978 [27, 28]
4	Magora *et al*, 1980 [11]	Wang, 1978 [14] Wang, 1979 [8] Behar *et al*, 1979 [9] Cousins *et al*, 1979 [10]	Snyder, 1977 [23, 24] Yaksh *et al*, 1977 [26] Yaksh *et al*, 1978 [30]
5	Bromage *et al*, 1980 [12]	Wang, 1979 [8] Behar *et al*, 1979 [9] Cousins *et al*, 1979 [10] Samii *et al*, 1979 [15]	Calvillo *et al*, 1974 [31] Kitahata *et al*, 1974 [32] LeBars *et al*, 1974 [33] Pert *et al*, 1976 [20] Yaksh, 1978 [34]

**Note: **^a^ This article has information on the preliminary observation on the analgesic effect of intrathecal morphine in humans.

## ABBREVIATION

PI: Popularity Index

## References

[1] Yaksh T.L. (1981). Spinal opiate analgesia: Characteristics and principles of action.. Pain.

[2] Rawal N. (1999). Epidural and spinal agents for postoperative analgesia.. Surg. Clin. North Am..

[3] Kitahata L.M., Collins J.G. (1981). Spinal action of narcotic analgesics.. Anesthesiology.

[4] Cousins M.J., Mather L.E. (1984). Intrathecal and epidural administration of opioids.. Anesthesiology.

[5] Merton RK (1957). Priority in scientific discovery: A chapter in the sociology of science.

[6] Stevens M. (2003). The role of the priority rule in science.. J. Philos..

[7] Vale R.D., Hyman A.A. (2016). Priority of discovery in the life sciences.. eLife.

[8] Wang J.K., Nauss L.A., Thomas J.E. (1979). Pain relief by intrathecally applied morphine in man.. Anesthesiology.

[9] Behar M., Magora F., Olshwang D., Davidson J.T. (1979). Epidural morphine in treatment of pain.. Lancet.

[10] Cousins M.J., Mather L.E., Glynn C.J., Wilson P.R., Graham J.R. (1979). Selective spinal analgesia.. Lancet.

[11] Magora F., Olshwang D., Eimerl D. (1980). Observations on extradural morphine analgesia in various pain conditions.. Br. J. Anaesth..

[12] Bromage P.R., Camporesi E., Chestnut D. (1980). Epidural narcotics for postoperative analgesia.. Anesth. Analg..

[13] Wang J.K. (1977). Analgesic effect of intrathecally administered morphine.. Reg. Anesth..

[14] Wang J.K. (1978). Pain relief by intrathecal injection of serotonin or morphine.. Ann. Anesthesiol. Fr..

[15] Samii K, Feret J, Harari A, Viars P. Selective spinal analgesia.. Lancet.

[16] Scott D.B., McClure J. (1979). Selective epidural analgesia.. Lancet.

[17] Glynn C.J., Mather L.E., Cousins M.J., Wilson P.R., Graham J.R. (1979). Spinal narcotics and respiratory depression.. Lancet.

[18] Liolios A., Andersen F.H. (1979). Selective spinal analgesia.. Lancet.

[19] Wang J.K. (1977). Antinociceptive effect of intrathecally administered serotonin.. Anesthesiology.

[20] Pert C.B., Kuhar M.J., Snyder S.H. (1976). Opiate receptor: Autoradiographic localization in rat brain.. Proc. Natl. Acad. Sci..

[21] Yaksh T.L., Rudy T.A. (1976). Analgesia mediated by a direct spinal action of narcotics.. Science.

[22] Yaksh T.L., Huang S.P., Rudy T.A. (1977). The direct and specific opiate-like effect of met5-enkephalin and analogues on the spinal cord.. Neuroscience.

[23] Snyder S.H. (1977). Opiate receptors in the brain.. N. Engl. J. Med..

[24] Snyder S.H. (1977). Opiate receptors and internal opiates.. Sci. Am..

[25] Yaksh T.L., Yeung J.C., Rudy T.A., Plant R. (1976). Evidence for an analgetic action of morphine in spinal cord.. Fed Am Soc Exp Biol.

[26] Yaksh T.L., Rudy T.A. (1977). Studies on the direct spinal action of narcotics in the production of analgesia in the rat.. J. Pharmacol. Exp. Ther..

[27] Yaksh T.L., Frederickson R.C., Huang S.P., Rudy T.A. (1978). In vivo comparison of the receptor populations acted upon in the spinal cord by morphine and pentapeptides in the production of analgesia.. Brain Res..

[28] Yaksh T.L., Wilson P.R. (1979). Spinal serotonin terminal system mediates antinociception.. J. Pharmacol. Exp. Ther..

[29] Yaksh T.L., Wilson P.R., Kaiko R.F., Inturrisi C.E. (1979). Analgesia produced by a spinal action of morphine and effects upon parturition in the rat.. Anesthesiology.

[30] Yaksh T.L., Rudy T.A. (1978). Narcotic analgestics: CNS sites and mechanisms of action as revealed by intracerebral injection techniques.. Pain.

[31] Calvillo O., Henry J.L., Neuman R.S. (1974). Effects of morphine and naloxone on dorsal horn neurones in the cat.. Can. J. Physiol. Pharmacol..

[32] Kitahata L.M., Kosaka Y., Taub A., Bonikos K., Hoffert M. (1974). Lamina-specific suppression of dorsal-horn unit activity by morphine sulfate.. Anesthesiology.

[33] Le Bars D., Menetrey D., Conseiller C., Besson J.M. (1974). Comparaison chez le chat spinal et le chat.. CR Acad Sci.

[34] Yaksh T.L. (1978). Analgetic actions of intrathecal opiates in cat and primate.. Brain Res..

[35] Bier A. (1889). Versuche uber cocainisirung des ruckenmarkes.. Dtsch Z Chir.

[36] Racoviceanu-Pitesti N. (1900). Anesthesia generala prin cocaina.. Rev. Chir..

[37] Matsuki A. (1983). Nothing new under the sun-a Japanese pioneer in the clinical use of intrathecal morphine.. Anesthesiology.

[38] Benedetti C. (1987). Intraspinal analgesia: A historical overview.. Acta Anaesthesiol. Scand. Suppl..

[39] Brill S., Gurman G.M., Fisher A. (2003). A history of neuraxial administration of local analgesics and opioids.. Eur. J. Anaesthesiol..

[40] Morgan M. (1989). The rational use of intrathecal and extradural opioids.. Br. J. Anaesth..

[41] Magora F. (2004). Historical data on the neuraxial administration of opioids.. Eur. J. Anaesthesiol..

[42] Winnie A.P. (1980). Epidural and intrathecal opiates. New uses for old drugs.. Anesthesiol. Rev..

[43] Vlassakov K.V., Kissin I. (2014). Scientometrics of anesthetic drugs and their techniques of administration, 1984-2013.. Drug Des. Devel. Ther..

[44] Hurley R.W., Murphy J.D., Wu C.L., Miller R.D. (2020). Acute postoperative pain.. Miller’s Anesthesia..

